# Impact of Aetiological Treatment on Conventional and Multiplex Serology in Chronic Chagas Disease

**DOI:** 10.1371/journal.pntd.0001314

**Published:** 2011-09-06

**Authors:** Rodolfo Viotti, Carlos Vigliano, María Gabriela Álvarez, Bruno Lococo, Marcos Petti, Graciela Bertocchi, Alejandro Armenti, Ana María De Rissio, Gretchen Cooley, Rick Tarleton, Susana Laucella

**Affiliations:** 1 Chagas Disease and Heart Failure Section, Cardiology Department, Hospital Eva Perón, San Martín, Buenos Aires, Argentina; 2 Instituto Nacional de Parasitología “Dr. Mario Fatala Chaben”, Buenos Aires, Argentina; 3 Center for Tropical and Emerging Global Diseases and Department of Cellular Biology, University of Georgia, Athens, Georgia, United States of America; National Institutes of Health, United States of America

## Abstract

**Background:**

The main criterion for treatment effectiveness in Chagas Disease has been the seronegative conversion, achieved many years post-treatment. One of the main limitations in evaluating treatment for chronic Chagas disease is the lack of reliable tests to ensure parasite clearance and to examine the effects of treatment. However, declines in conventional serological titers and a new multiplex assay can be useful tools to monitor early the treatment impact.

**Methodology/Principal Findings:**

Changes in antibody levels, including seronegative conversion as well as declines in titers, were serially measured in 53 benznidazole-treated and 89 untreated chronic patients in Buenos Aires, Argentina with a median follow-up of 36 months. Decrease of titers (34/53 [64%] treated vs. 19/89 [21%] untreated, p<0.001) and seronegative conversion (21/53, [40%] treated vs. 6/89, [7%] untreated, p<0.001) in at least one conventional serological test were significantly higher in the benznidazole-treated group compare with the untreated group. When not only complete seronegative conversion but also seronegative conversion on 2 tests and the decreases of titers on 2 or 3 tests were considered, the impact of treatment on conventional serology increased from 21% (11/53 subjects) to 45% (24/53 subjects). A strong concordance was found between the combination of conventional serologic tests and multiplex assay (kappa index 0.60) to detect a decrease in antibody levels pos-treatment.

**Conclusions/Significance:**

Treatment with benznidazole in subjects with chronic Chagas disease has a major impact on the serology specific for *T. cruzi* infection in a shorter follow-up period than previously considered, reflected either by a complete or partial seronegative conversion or by a significant decrease in the levels of *T. cruzi* antibodies, consistent with a possible elimination or reduction of parasite load.

## Author Summary

The main criterion for treatment effectiveness in Chagas Disease has been the seronegative conversion of previously reactive serology, generally achieved many years post-treatment. The lack of reliable tests to ensure parasite clearance and to examine the effect of treatment is the main difficulty in evaluating treatment for chronic Chagas disease. Decreases of conventional and non-conventional serological titers can be useful tools to monitor the early impact of treatment. We serially measured changes in antibody levels, including seronegative conversion as well as declines in titers in 53 benznidazole-treated and 89 untreated chronically *T. cruzi*-infected subjects. Seronegative conversion as well as decreases of titers was significantly higher in treated compared with untreated patients. A strong concordance was found between decreases of titers of conventional and non-conventional serologic tests post-treatment, reaffirming the findings. When seronegative conversion plus decreases of titers were considered altogether, the impact of treatment was higher, in a shorter follow-up period than previously considered. New tools for monitoring the effectiveness of treatment of chronic Chagas disease are necessary, and the results showed in this study is a contribution to researchers and physicians who assist patients suffering from this disease.

## Introduction

The chronic form of Chagas disease, now considered to be globalized is prevalent in Latin America as well as in countries where *T. cruzi*-infection is not endemic [1]–[3]. In Argentina, *T. cruzi* infection is diagnosed by the conventional serologic tests indirect immunofluorescence assay (IFI), indirect hemagglutination (IHA) and ELISA that measure the level of circulating antibodies against *Trypanosoma cruzi* antigenic components (generally intact or whole parasite lysates), with 2 positive tests out of the 3 performed being required to confirm *T. cruzi*-infection [4]. This ‘best 2 out of 3’ approach is standard for diagnosis of *T. cruzi* infection in most endemic countries.

Even though long term follow-up of chronic Chagas disease patients treated with benznidazole showed that specific chemotherapy can prevent the progression of the heart-related pathology of Chagas disease in several [5]–[7], but not all studies [8], a wider use of the available drugs has not been achieved. One of the main limitations in evaluating treatment for chronic Chagas disease is the lack of reliable tests to ensure parasite clearance and to evaluate the impact of treatment on the evolution of lesions in target tissues. The main criterion for “cure” has been the conversion to negative serology on all tests performed [9]. However, this result is often not observed until 8 to10 years post-treatment and then only in approximately 15% of treated adult subjects [5]–[7], [10]. Clearly, the identification of better surrogate markers of parasite load decrease or complete parasite elimination is needed.

We have recently shown that changes in *T. cruzi*–specific T cell responses and in antibody responses to individual parasite proteins as determined using a multiplex assay can be used as early and effective predictors of the impact of drug treatment in human Chagas disease [11]. In addition, several studies have shown that a fall in conventional serological titers might be also useful to indicate treatment impact [5], [7], [9], [12]. However, these protocols have not been exhaustively explored in short post-treatment follow-up periods. In this study, the changes in antibody levels specific for *T. cruzi* were measured by conventional serologic assays and by a new multiplex assay during a relatively short follow-up period after treatment with benznidazole in chronically *T. cruzi* infected subjects.

## Methods

### Subjects

Three hundred and twenty eight patients assisted in the first consultation at the Health Care Section for Chagas disease at the Hospital Eva Perón, Buenos Aires, Argentina were prospectively evaluated between 2004 and 2009. For inclusion in the study, a new serological evaluation by IFI, IHA and ELISA assays was conducted at the "Instituto Nacional de Parasitología “Dr. Mario Fatala Chaben”, the National Center of Reference for diagnosis of *T. cruzi* infection in Argentina. Of these 328 subjects who had a previous positive serology for *T. cruzi* in primary health care centers, 37 were excluded based upon negative tests at the Instituto Nacional de Parasitología “Dr. Mario Fatala Chaben”. Fifteen patients with discordant serology, 2 patients with associated coronary artery disease, 1 patient with HIV co-infection, and 1 patient living in an endemic area without vector control were also excluded from the present study. Adult individuals older than 21 years of age with 3 positive serological tests for *T. cruzi* infection, without heart disease (Group 0) or with mild heart disease (Group I), and serological follow-up of at least 24 months were included in this study. One hundred and thirty patients did not achieve the minimum follow-up period and were excluded for further analysis.

### Intervention

Aetiological treatment with benznidazole was offered to all patients, following the recommendations of the national and international guidelines for Chagas disease [13]–[15]. Benznidazole was administered at a dose of 5 mg/kg/day for 30 days. According to the principle of intention to treat, all patients, including those who did not complete the 30 day-treatment period (17%), were considered in the treated group.

### Measurements and study variables

A baseline electrocardiogram (ECG) and a 2-D echocardiogram were performed to stratify the patients according to a modified Kuschnir classification as follow: Group 0 has positive serology on 3 conventional serological tests, normal ECG and normal echocardiogram; and Group I includes subjects with positive serology on 3 tests, abnormal ECG (arrhythmias or conduction disturbances) and echocardiogram without left ventricular dilation or dysfunction [16]. Patients in group II (with dilation and left ventricular dysfunction) and in group III (with heart failure) were not included due to the low number of subjects with these conditions.

#### Conventional serology

Serology for *T. cruzi* infection was performed on admission and repeated yearly. The serological tests were standardized at the "Instituto Nacional de Parasitología “Dr. Mario Fatala Chaben" where sera samples were received blinded for treatment and for the clinical stage of the patients. IHA and IFI assays were reported as reactive from sequential ½ titer dilutions between 1/32 and 1/256. ELISA test was considered positive when mean absorbance at 490 nm was higher than the cut-off value of 0.200.

The magnitude in serological titer reduction at different time points post treatment respect to titers at baseline was calculated for each subject. Decreases in at least 1 titer dilution on IHA and IFI (reduction of 50%) and at least a 30% reduction on ELISA assays compared with baseline values were considered as significant changes. Serological variations were evaluated annually, while the latest serological data available was considered for global analysis. The ELISA cut-off of 30% was defined according to the analysis of sensitivity/specificity and positive likelihood ratios (sensitivity/1-specificity) to achieve titer decreases in benznidazole-treated subjects that were significantly different compared with those in the untreated group. The minimum follow-up period to detect a decrease of titers or seronegative conversion respective to baseline serology was also determined.

Conversion to negative serology was confirmed when titers were lower than 1/32 for IHA and IFI and the mean absorbance at 490 nm in ELISA assays was under 0.200. Because the standard for determining a diagnosis of chronic Chagas disease as a positive result is based on at least 2 tests out of 3 conventional serological tests, we considered the conversion to negative serology on 2 or 3 tests as an indicator of cure.

#### Multiplex Serologic Assay

Serum specimens of 44 patients (32 treated and 12 untreated patients) out of the 142 patients evaluated by conventional serology were screened for antibodies reactive to a panel of 14 recombinant *T. cruzi* proteins in a Luminex-based format, as previously described [17]. Sampling was performed prior to treatment and then, 2 and 6 months, and annually after treatment. Serological responses to each individual *T. cruzi* protein were considered to have decreased during the study period if the mean fluorescence intensity for at least one recombinant protein decreased≥40% relative to that of the time 0 (pretreatment) sample assayed concurrently. The multiplex cut-off of 40% was defined according to the analysis of sensitivity/specificity and positive likelihood ratios (sensitivity/1-specificity) to achieve titer decreases in benznidazole-treated subjects that were significantly different compared with those recorded in untreated subjects.

### Statistical analysis

Continuous measures were expressed as mean and standard deviation or median and interquartile range of 25–75%, whereas dichotomous variables were expressed as a result/total and percentage. Chi square test or Fischer's exact test was performed to evaluate differences between discrete variables, whereas Student t test was applied in the study of continuous numerical variables. A Spearman rank correlation test and kappa index were applied to compare the decreases in antibody levels specific for *Trypanosoma cruzi* after treatment with benznidazole by the Multiplex and conventional serological tests.

Sensitivity, specificity and positive likelihood ratios (sensitivity/1-specificity) for detecting decreases of titers were determined for the Multiplex assay, and for the 3 conventional serological tests taken together or each serological taken separately. For the multivariate analysis, the logistic regression model was used, including all variables with significance (p<0.05) in the Chi square test and Student t test, as well as gender and years of residence in endemic areas for being considered clinically relevant.

The relative risk with a 95% confidence interval and the number needed to treat to achieve a change in post treatment serology was determined. All statistical analysis was performed using Analytical Software Statistix 8.0. The protocol was approved by the review Boards of the Eva Perón Hospital. Signed informed consent was obtained from all individuals before inclusion in the study.

## Results

### Variations in conventional serological tests

We included 142 patients, 85 female and 57 male, mean ages 42.2±8.4 years. Fifty three patients (37%) were treated with benznidazole while 89 remained untreated (63%). Patients who agreed to receive treatment were marginally younger (mean age treated group = 39.7±8.4 vs. mean age untreated group = 43.7±8.1, p = 0.006) and had a higher likelihood of ECG abnormalities compared with untreated subjects (14/53, 26% of treated patients vs. 4/89, 4% of untreated patients, p<0.001). The median time and interquartile range (25–75%) of follow-up was 36 months (32–48) for the entire study population [48 months (36–60) for treated and 36 months (27.5–48) for untreated patients, p<0.001]; sex (male in treated group = 23/53, 43% vs. male untreated group = 34/89, 38%, p = 0.54) and years of residence in endemic areas (treated = 14.4±9.9 vs. untreated = 14.5±9.1, p = 0.94) were not significantly different between treated and untreated patients.

**Figure 1:**
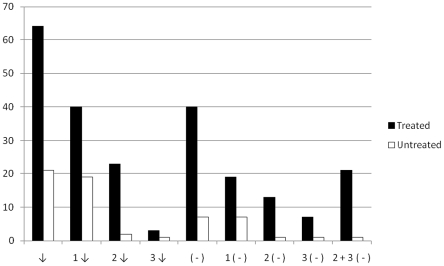
Percentages of titers decrease and seronegative conversion in treated and untreated chronically *T. cruzi*-infected subjects^(a)^. (a) The latest available serological data at the end of the study was considered for analysis. ↓ = decrease of titers on at least 1 test; 1↓  =  titer decrease on 1 test; 2 ↓  =  titer decrease on 2 tests; 3 ↓  =  titer decrease on 3 tests; (−)  =  seronegative conversion on at least 1 test; 1 (−)  =  seronegative conversion on 1 test; 2 (−)  =  seronegative conversion on 2 tests; 3 (−)  =  seronegative conversion on 3 tests; 2+3 (−)  =  seronegative conversion on 2 or 3 tests.

In order to evaluate treatment success, seronegative conversion and also decreases of titers in at least one test were determined in the present study. The frequency of decrease in titers as well as the conversion to negative results on one or more conventional serologic tests was significantly greater in the treated patient group compared with untreated individuals (Table 1 and Figure 1). Complete seroconversion was found in 4 out of 53 (8%) while seronegative conversion on 2 tests was observed in 7 out of 53 (13%) treated subjects. Because the standard for determining a diagnosis of *T. cruzi* infection as a positive result is based on at least 2 out of 3 conventional serological tests, we extended the criteria of cure to the seronegative conversion on 2 tests, as well. Thus, conversion to negative serology on 2 or 3 tests was observed in 11 out of the 53 treated subjects (21%).

**Table 1:** Impact of treatment with benznidazole on conventional serology in chronic Chagas disease.

Serological changes (a)	Treated(b) (N = 53)	Untreated (b) (N = 89)	p	Relative risk (CI 95%)	Number needed to treat
**Decrease of titers on at least 1 serological test**	34 (64)	19 (21)	<0.001	3.00 (1.92–4.69)	2
**Decrease of titers on 1 serological test**	21 (40)	17 (19)	0.007		
**Decrease of titers on 2 serological tests**	12 (23)	2 (2)	<0.001		
**Decrease of titers on 3 serological tests**	1 (2)	0 (0)	0.19		
**Seronegative conversion on at least 1 test**	21 (40)	6 (7)	<0.001	8.91 (3.81–20.85)	3
**Seronegative conversion on 1 test**	10 (19)	6 (7)	0.03		
**Seronegative conversion on 2 tests**	7 (13)	0 (0%)	<0.001		
**Seronegative conversion on 3 tests**	4 (8)	0 (0%)	0.009		
**Seronegative conversion on 2 and 3 tests (Indicator of cure)**	11 (21)	0 (0%)	<0.001	18.47 (2.45–130.06)	5
**Impact of treatment ** (c)	24 (45)	2 (2)	<0.001	20.1 (4.96–81.87)	2

(a) The latest available serological data at the end of the study was considered for analysis.

(b) The figures represent the number of subjects with the indicated changes out of the total evaluated (%).

(c) Includes subjects with decrease of titers or seronegative conversion on 2 or 3 tests.

IHA titers decreased in 22 out of 53 (41%) treated patients and in 8 out of 89 (9%) untreated subjects, p<0.001. Mean absorbance by ELISA decreased in 18 out of 53 (34%) treated and in 6 out of 89 (7%) untreated subjects, p<0.001. Finally, a decline in titers by IFI assays was observed in 18 out of 53 (34%) treated and in 7 out of 89 (8%) untreated subjects, p<0.001. In treated subjects, the median follow-up period to detect a decline in antibody levels was 27 months (interquartile range 25 to 75% 16.5–38.5), while a median of 24 months (interquartile range 25 to 75% 21–27.7) was required to identify seroconversion on 2 or 3 tests.

The aetiological treatment with benznidazole was the only variable that correlated with the decline of titers or seronegative conversion as determined by multivariate analysis (Table 2). None of the treated or untreated patients showed progression of the heart disease (new ECG changes or evolution to more severe clinical stages) during the follow-up period of this study.

**Table 2:** Logistic regression for decrease of titers and seronegative conversion on conventional serological assays in *T.cruzi*-infected subjects.

	Decrease of titers		Seronegative conversion	
	Coefficient β	P	Coefficient β	P
Age	0.03	0.26	0.019	0.55
Gender	0.20	0.64	0.34	0.48
Group 0 (without heart disease)	1.11	0.094	0.22	0.73
Years of residence in endemic area	0.008	0.70	0.04	0.08
Treatment with benznidazole	2.87	<0.001	2.34	<0.001

Altogether, these findings show that taking into account either conversion to negative serology on 2 or 3 tests and decreases of titers on 2 or 3 tests, the impact of treatment on conventional serology might increase to 45%.

### Comparison of conventional serology and multiplex analysis

We have previously shown that treatment with benznidazole decreased antibody responses specific for a group of *T. cruzi* recombinant proteins as detected by a multiplex assay [11]. Decreases of antibody titers by conventional serology, as defined in Material and Methods, and by multiplex analysis were compared in a subset of individuals, 32 benznidazole-treated and 12 untreated subjects (50% male/female with a mean age of 40.7±7.7) during the follow-up period of the study. Seventy five percent of the subjects assessed showed similar trends with respect to the combined 3 conventional serologic tests and the multiplex assay (simple concordance = 75%; Kappa index = 0.60). In addition, a positive correlation was found between decreases in antibody titers measured by conventional serological tests and the multiplex (r = 0.59, p<0.001). Overall, 26/32 treated subjects (81%) and 1/12 untreated subjects (8%) showed a decline in antibody responses considering both conventional serological tests and the multiplex assay (Table 3). Although the overall results in this set of subjects were similar using the multiplex and combined conventional serological tests, the multiplex test was superior in detecting serological changes when compared to each conventional serologic test separately (i.e. 7 subjects showed titer decreases by multiplex while no alterations were recorded by ELISA, Table 3).

**Table 3:** Reduction of antibody levels by Multiplex and conventional serology after treatment with benznidazole.

	Reduction of titers								
ID	Multiplex			ELISA		IHA		IFI	
	% a	N° b	Time of titer decrease (mo) c	% a	Time of titer decrease (mo) c	% a	Time of titer decrease (mo) c	% a	Time of titer decrease (mo) c
PP 01	54–71	4/6	36	-		-		50	6
PP 03	41–58	5/6	24	-	-	-	-	-	-
PP 06	-	0/3	-	-	-	-	-	-	-
PP 16	-	0/8	-	40	48	-	-	50	48
PP 18	41–60	7/11	12	-	-	75	25	-	-
PP 21	-	0/3	-	-	-	-	-	-	-
PP 24	41–68	3/5	6	60	24	-	-	50	7
PP 26	-	0/2	-	-	-	-	-	-	-
PP 31	40–47	2/3	12	-	-	-	-	-	-
PP 44	41	1/5	31	40	37	-	-	-	-
PP 55	62–66	3/7	24	30	24	-	-	-	-
PP 62	-	0/2	-	-	-	-	-	-	-
PP 67	-	0/1	-	30	24	50	63	-	-
PP 83	-	NR d	-	-	-	-	-	50	48
PP 85	41–63	4/6	14	34	14	-	-	75	14
PP 93	47–53	3/7	24	60	36	-	-	50	52
PP 100	64	1/1	12	-	-	75	24	50	60
PP 113	57–69	4/5	12	-	-	75	24	50	36
PP 114	56	1/8	36	30	24	-	-	-	-
PP 115	66–85	7/8	24	30	36	-	-	-	-
PP 117	-	NR d	-	-	-	50	12	50	12
PP 118	40	1/3	36	-	-	-	-	50	48
PP 120	41	1/3	26	50	27	50	27	-	-
PP 137	-	0/9	-	-	-	-	-	-	-
PP 149	41–43	2/6	36	-	-	50	60	50	72
PP 164	48–96	7/7	2	-	-	-	-	-	-
PP 178	45–46	3/3	48	-	-	50	12	-	-
PP 179	51–59	4/6	36	-	-	87	6	-	-
PP 186	62–64	3/3	6	-	-	-	-	-	-
PP 198	40	1/4	24	-	-	-	-	50	48
PP 384	-	-	-	-	-	-	-	-	-
PP 385	57–92	6/8	2	30	24	-	-	-	-

a Range percentage of reduction in antibody levels compared with baseline at the end of follow-up.

b Number of proteins with≥40% decreases in reactivity after treatment out of the total reactive proteins prior to treatment.

c Time of follow-up where reactivity decrease was observed.

d NR No reactive prior to treatment. (−) Changes in serological titers were not observed during follow-up.

## Discussion

This study shows the unquestionable impact of aetiological treatment with benznidazole on conventional and multiplex serology for *T cruzi*-infection by three years postreatment. A significant proportion of treated patients reached the extended criteria of cure (conversion to seronegative on 2 or 3 tests) presented herein, while an even larger number of subjects showed declines in the titers on one or more tests compared with untreated adult subjects in this relatively short follow-up span. The strong correlation between the combined 3 conventional serologic tests and the multiplex assay, that measures antibody levels to individual recombinant proteins, also provides support for the impact of benznidazole treatment on chronic *T. cruzi* infection.

Cure rates of 8–40% in a 10–20 year-average of follow-up have been reported in the late chronic phase of adult patients who received etiological treatment for *T. cruzi* infection [18]. Work from our group showed a conversion to negative serology in only 15% of benznidazole-treated subjects after a 10-year follow-up study with serological tests performed every 3-year interval of time [6]. It is noteworthy that in the present study we were able to detect a 7% rate of seronegative conversion in less than half the time of our former studies. The more frequent sampling might likely improve the detection of both seronegative conversion and decreases in serological titers. In agreement with our findings, a recent publication has also described complete seroconvertion of 5% in a 3-year follow-up study after treatment with benznidazole [19]. Because the standard for determining a diagnosis of *T. cruzi* infection is based on at least 2 tests out of 3 conventional serological tests performed, we considered the conversion to negative serology on 2 or 3 tests as a possible indicator of cure. Although, spontaneous cure is a rare event documented in long-term untreated *T. cruzi*-infected subjects [20], [21], in this short-term study we have not recorded any case.

A reduction range of 28% to 34% in conventional serological titers in benznidazole-treated subjects was also demonstrated [7]. Likewise, declines in serological titers after treatment with benznidazole in chronically *T. cruzi*-infected subjects were shown by using modified conventional serological tests with recombinant antigens [12].

Herein, we found that a significant proportion of benznidazole-treated subjects showed changes in serological titers, from complete or partial conversion to negative serology by conventional tests to decreases in antibody levels determined by not only by conventional serological tests but also by the multiplex assay. The most plausible explanation for these observations is that those subjects who showed a decline of titers have achieved a reduction in parasite load or even complete parasite elimination, but a longer observation time is required to achieve complete seroconversion to negative. Concurring with this notion, we had previously documented that decreases in serological responses in benznidazole-treated subjects were associated with better clinical outcomes compared with untreated subjects, even when complete conversion to negative serology had not been achieved [5], [22]. More recently, we have shown that in the majority of benznidazole-treated subjects *T. cruzi*-specific IFN-γ- producing T cells significantly decreased or became negative along with decreases in antibody responses measured by the multiplex assay in a 5-year follow-up study, other likely indicators of treatment efficacy [11], [23]. Likewise, a reduction in parasitemia, as determined by PCR, was recently documented in chronic patients treated with benznidazole in a one year follow-up study, although serological changes were not reported in this short-term period. In this same study, PCR shifted to positive in around 10% of treated subjects showing that parasites were not completely eradicated in these subjects [24].

Serum antibodies have the capacity to be maintained for prolonged periods even in the absence of antigen making it challenging to correlate treatment efficacy with the measurement of changes in serology [25]. In this regard, antibody responses to viruses as varicella-zoster, measles and mumps in humans have been shown to be maintained for more than 50 years (in the case of smallpox even longer) [26]. Memory B-cell-independent antibody production by long-lived plasma cells has been proposed as the main mechanism to maintain long-term humoral immunity [27], [28]. The lifespan of long-lived plasma cells depends on external survival signals received in survival niches, mainly located in the bone marrow, although a small proportion of long-lived plasma cells persist in the spleen of both humans and mice [29]. However, under chronic inflammatory conditions, plasma cells are also found in inflamed tissues [29]. Therefore, a decrease in parasite burden in benznidazole-treated subject might be responsible for the elimination of survival niches in parasitized tissues limiting the lifespan of long-lived plasma cells with the subsequent decline in antibodies titers.

According to our and other author's findings, it can be proposed the notion of “impact of treatment” to extend the concept of successful treatment, by considering not only complete seroconversion but also conversion to negative serology in 2 tests, plus the decreases of titers in 2 or 3 conventional serological tests or as measured by the multiplex assay. Applying, the criteria proposed herein, when decreases of titers and negative seroconversion on 2 and 3 serological tests were taken into account (impact of treatment, Table 1), the effectiveness of treatment is significantly higher, reaching close to 45%.

Of note, the multiplex assay was able to detect the same level of decreases in serological titers as the overall decreases detected by the 3 conventional tests in combination, indicating the potential of this novel technique as a single and reproducible marker of treatment impact. Furthermore, the multiplex assay detected serological decreases following treatment with benznidazole in several cases in which conventional serological tests did not vary and some subjects showed decreases by multiplex as soon as 2–6 months post-treatment, as well.

While the main purpose of aetiological treatment is the complete elimination of *T. cruzi*, a diminution in parasite load might be relevant from the clinical standpoint. The available data support the conclusion that Chagas disease is the result of the failure of the immune system to completely clear this persistent infection, and the effect of decades of immune assault [30], [31]. One of the possible mechanisms is that the constant antigen stimulation might lead to a process of immune exhaustion which in turn might damper the ability of the immune system to control the infection with the subsequent disease progression [32], [33]. However, other regulatory pathways might also be involved in parasite control during the chronic infection [34]–[36]. Therefore, even a reduction in parasite load might restore, to some extent, the functionality of the immune system to keep the parasite under control with stable clinical conditions [37], [38].

Another scenario that emerges from the results presented herein is that completely unchanged serological findings after 3 years of treatment might be suggestive of treatment failure, while a decline of titers may indicate that a curative response is ongoing. Future studies might confirm whether unchanged conventional or multiplex serological assays or unaltered *T cruzi*-specific T cell responses following treatment reflect treatment failure, which might support the possibility of a new round of treatment, or the use of alternative drug treatments [39], [40].

The main limitation of this study is the non-randomized design of it, and thus rendering the findings of lesser strength compared with a randomized design.Our results should be also extrapolated with caution due to the possibility that diverse *T cruzi* lineages might have different susceptibility to benznidazole and thus treatment effectiveness may vary among endemic regions of Latin-American [41].

In summary, treatment with benznidazole in subjects with chronic Chagas disease has a major impact on the serology specific for *T. cruzi* infection in a shorter follow-up period than previously considered, reflected either by a complete negativization or by a significant decrease in the levels of *T. cruzi* antibodies, consistent with a possible elimination or reduction of parasite load.

## References

[1] Moncayo A, Silveira AC (2009). Current epidemiological trends for Chagas disease in Latin America and future challenges in epidemiology, surveillance and health policy.. Mem Inst Oswaldo Cruz.

[2] Gascón J, Albajar P, Cañas E, Flores M, Gómez i Prat J (2007). Diagnóstico, manejo y tratamiento de la cardiopatía chagásica crónica en áreas donde la infección por Trypanosoma cruzi no es endémica.. Rev Esp Cardiol.

[3] Schmunis GA (2007). Epidemiology of Chagas disease in non-endemic countries: the role of international migration.. Mem Inst Oswaldo Cruz.

[4] World Health Organization (1991). Control of Chagas disease. Report of aWHO Expert Committee.. World Health Organ Tech Rep Ser.

[5] Viotti R, Vigliano C, Armenti H, Segura E (1994). Treatment of chronic Chagas' disease with benznidazole: clinical and serologic evolution of patients with long-term follow-up.. Am Heart J.

[6] Viotti R, Vigliano C, Lococo B, Bertocchi G, Petti M (2006). Long-term cardiac outcomes of treating chronic Chagas disease with benznidazole versus no treatment.. Ann Intern Med.

[7] Fabbro D, Streiger M, Arias E, Bizal ML, Del Barco M (2007). Tripanocide treatment among adults with chronic Chagas disease living in Santa Fe city (Argentina), over a mean follow-up of 21 years: parasitological, serological and clinical evolution.. Rev Soc Bras Med Trop.

[8] Lauria-Pires L, Braga MS, Vexenat AC, Nitz N, Simões-Barbosa A (2000). Progressive chronic Chagas heart disease ten years after treatment with anti-*Trypanosoma cruzi* nitroderivatives.. Am J Trop Med Hyg.

[9] Cançado JR (1999). Criteria of Chagas Disease Cure.. Mem Inst Oswaldo Cruz.

[10] Gallerano RR, Sosa RR (2000). Interventional study in the natural evolution of Chagas disease. Evaluation of specific antiparasitic treatment. Retrospective-prospective study of antiparasitic therapy.. Rev Fac Cien Med Univ Nac Cordoba.

[11] Laucella SA, Mazliah DP, Bertocchi G, Alvarez MG, Cooley G (2009). Changes in Trypanosoma cruzi-specific immune responses after treatment: surrogate markers of treatment efficacy.. Clin Infect Dis.

[12] Sánchez Negrette O, Sánchez Valdez FJ, Lacunza CD, García Bustos MF, Mora MC (2008). Serological evaluation of specific-antibody levels in patients treated for chronic Chagas' disease.. Clin Vaccine Inmunol.

[13] Sosa Estani S, Segura E (1999). Tratamiento de la infección por T cruzi en fase indeterminada. Experiencia y normatización actual en la Argentina.. Medicina (Buenos Aires).

[14] WHO Technical Report Series 905 (2002). Control of Chagas disease, Aetiological treatment.. Geneva, Switzerland,.

[15] Bern C, Montgomery SP, Herwaldt BL, Rassi A, Marin-Neto JA (2007). Evaluation and treatment of Chagas disease in the United States: a systematic review.. JAMA.

[16] Kuschnir E, Sgammini H, Castro R, Evequoz C, Ledesma R (1985). Valoración de la función cardiaca por angiografía radioisotópica, en pacientes con cardiopatía chagásica crónica.. Arq Bras Cardiol.

[17] Cooley G, Etheridge RD, Boehlke C, Bundy B, Weatherly DB (2008). High Throughput Selection of Effective Serodiagnostics for Trypanosoma cruzi infection.. PlosNTDS;.

[18] Sosa-Estani S, Viotti R, Segura E (2009). Therapy, diagnosis and prognosis of chronic Chagas disease: insight gained in Argentina.. Mem Inst Oswaldo Cruz.

[19] Fernandes CD, Tiecher FM, Balbinot MM, Liarte DB, Scholl D (2009). Efficacy of benznidazole treatment for asymptomatic chagasic patients from state of Rio Grande do Sul evaluated during a three years follow-up.. Mem Inst Oswaldo Cruz.

[20] Francolino SS, Antunes AF, Talice R, Rosa R, Selanikio J (2003). New evidence of spontaneous cure in human Chagas' disease.. Rev Soc Bras Med Trop.

[21] Pinto Dias JC, Dias E, Filho OM, Vitelli-Avelar D, Correia D (2008). Further evidence of spontaneous cure in human Chagas disease.. Rev Soc Bras Med Trop.

[22] Viotti R, Vigliano C, Lococo B, Bertocchi G, Petti M (2005). Clinical predictors of chronic chagasic myocarditis progression.. Rev Esp Cardiol.

[23] Bertocchi G, Álvarez MG, Pérez D, Armenti A, Viotti R (2008). Evaluación inmunológica del tratamiento con benznidazole en la enfermedad de Chagas crónica.. Rev Argent Cardiol.

[24] Murcia L, Carrilero B, Muñoz MJ, Asunción Iborra M, Segovia M (2010). Usefulness of PCR for monitoring benznidazole response in patients with chronic Chagas' disease: a prospective study in a non-disease-endemic country.. J Antimicrob Chemother.

[25] Siegrist CA, Aspinall R (2009). B-cell responses to vaccination at the extremes of age. Nat Rev Immunol..

[26] Amanna IJ, Carlson NE, Slifka MK (2007). Duration of humoral immunity to common viral and vaccine antigens.. N Engl J Med.

[27] Slifka MK, Ahmed R (1998). Long-lived plasma cells: a mechanism for maintaining persistent antibody production.. Curr Opin Immunol.

[28] Edwards JC, Szczepanski L, Szechinski J, Filipowicz-Sosnowska A, Emery P (2004). Efficacy of B-cell-targeted therapy with rituximab in patients with rheumatoid arthritis.. N Engl J Med.

[29] Radbruch A, Muehlinghaus G, Luger EO, Inamine A, Smith KG (2006). Competence and competition: the challenge of becoming a long-lived plasma cell.. Nat Rev Immunol.

[30] Tarleton R (2001). Parasite persistence in the aetiology of Chagas disease.. Int J Parasitol.

[31] Higuchi ML, Benvenuti LA, Reis MM, Metzger M (2003). Pathophysiology of the heart in Chagas disease: current status and new developments Cardiovascular Research.

[32] Laucella SA, Postan M, Martin D, Hubby Fralish B, Albareda MC (2004). Frequency of interferon- gamma -producing T cells specific for Trypanosoma cruzi inversely correlates with disease severity in chronic human Chagas disease.. J Infect Dis.

[33] Albareda M C, Laucella SA, Álvarez MG, Armenti AH, Bertocchi G (2006). Trypanosoma cruzi modulates the profile of memory CD8 T cells in chronic Chagas disease patients. Int.. Immunol.

[34] Fortes Araujo F, Silva Gomes J, Costa Rocha MO, Williams-Blangero S, Martins Pinheiro V, el al (2007). Potential role of CD4+CD25HIGH regulatory T cells in morbidity in Chagas disease.. Frontiers in Bioscience.

[35] Poncini CV, Giménez G, Pontillo CA, Alba-Soto CD, de Isola ELD (2010). Central role of extracellular signal-regulated kinase and Toll-like receptor 4 in IL-10 production in regulatory dendritic cells induced by *Trypanosoma cruzi.*. Molecular Immunology.

[36] da Matta Guedes PM, Gutierrez FR, Maia FL, Milanezi CM, Silva GK (2010). IL-17 produced during Trypanosoma cruzi infection plays a central role in regulating parasite-induced myocarditis.. PLoS Negl Trop Dis.

[37] Bustamante JM, Bixby LM, Tarleton RL (2008). Drug-induced cure drives conversion to a stable and protective CD8+ T central memory response in chronic Chagas disease.. Nat Med.

[38] Fernández MC, González Cappa SM, Solana ME (2010). Trypanosoma cruzi: immunological predictors of benznidazole efficacy during experimental infection.. Experimental Parasitology.

[39] Viotti R, Vigliano C (2007). Etiological treatment of chronic Chagas disease: neglected “evidence” by evidence-base medicine.. Expert Rev Anti Infect Ther.

[40] Araújo MSS, Martins-Filho OA, Pereira MES, Brener Z (2000). A combination of benznidazole and ketaconazole enhances efficacy of chemotherapy of experimental Chagas disease.. JAC.

[41] Higo H, Miura S, Horio M, Mimori T, Hamano S (2004). Genotypic variation among lineages of Trypanosoma cruzi and its geographic aspects.. Parasitology International.

